# Ly6C-high monocytes alleviate brain injury in experimental subarachnoid hemorrhage in mice

**DOI:** 10.1186/s12974-023-02939-y

**Published:** 2023-11-17

**Authors:** Huaijun Chen, Chaoran Xu, Hanhai Zeng, Zhihua Zhang, Ning Wang, Yinghan Guo, Yonghe Zheng, Siqi Xia, Hang Zhou, Xiaobo Yu, Xiongjie Fu, Tianchi Tang, Xinyan Wu, Zihang Chen, Yucong Peng, Jing Cai, Jianru Li, Feng Yan, Chi Gu, Gao Chen, Jingyin Chen

**Affiliations:** 1 Department of Neurosurgery, The Second Affiliated Hospital of Zhejiang University School of Medicine, Hangzhou, China; 2 Key Laboratory of Precise Treatment and Clinical Translational Research of Neurological Diseases, Hangzhou, China

**Keywords:** Subarachnoid hemorrhage, Inflammatory response, Immune response, Ly6C high monocyte

## Abstract

**Background:**

Subarachnoid hemorrhage (SAH) is an uncommon type of potentially fatal stroke. The pathophysiological mechanisms of brain injury remain unclear, which hinders the development of drugs for SAH. We aimed to investigate the pathophysiological mechanisms of SAH and to elucidate the cellular and molecular biological response to SAH-induced injury.

**Methods:**

A cross-species (human and mouse) multiomics approach combining high-throughput data and bioinformatic analysis was used to explore the key pathophysiological processes and cells involved in SAH-induced brain injury. Patient data were collected from the hospital (n = 712). SAH was established in adult male mice via endovascular perforation, and flow cytometry, a bone marrow chimera model, qPCR, and microglial depletion experiments were conducted to explore the origin and chemotaxis mechanism of the immune cells. To investigate cell effects on SAH prognosis, murine neurological function was evaluated based on a modified Garcia score, pole test, and rotarod test.

**Results:**

The bioinformatics analysis confirmed that inflammatory and immune responses were the key pathophysiological processes after SAH. Significant increases in the monocyte levels were observed in both the mouse brains and the peripheral blood of patients after SAH. Ly6C-high monocytes originated in the bone marrow, and the skull bone marrow contribute a higher proportion of these monocytes than neutrophils. The mRNA level of Ccl2 was significantly upregulated after SAH and was greater in CD11b-positive than CD11b-negative cells. Microglial depletion, microglial inhibition, and CCL2 blockade reduced the numbers of Ly6C-high monocytes after SAH. With CCR2 antagonization, the neurological function of the mice exhibited a slow recovery. Three days post-SAH, the monocyte-derived dendritic cell (moDC) population had a higher proportion of TNF-α-positive cells and a lower proportion of IL-10-positive cells than the macrophage population. The ratio of moDCs to macrophages was higher on day 3 than on day 5 post-SAH.

**Conclusions:**

Inflammatory and immune responses are significantly involved in SAH-induced brain injury. Ly6C-high monocytes derived from the bone marrow, including the skull bone marrow, infiltrated into mouse brains via CCL2 secreted from microglia. Moreover, Ly6C-high monocytes alleviated neurological dysfunction after SAH.

**Supplementary Information:**

The online version contains supplementary material available at 10.1186/s12974-023-02939-y.

## Introduction

Subarachnoid hemorrhage (SAH) is a type of stroke caused by the rupture of intracranial blood vessels, which results in blood flowing into the subarachnoid space [1]. SAH is mainly induced by the rupture of intracranial aneurysms [2] and has high mortality and disability rates [3]. The reported mortality rate is 25–50%, but this estimate does not fully account for patients who die before receiving medical care [1]. Approximately 50% of the surviving SAH patients suffer from long-term neurological deficits, such as hemiplegia and aphasia, and thus often need long-term rehabilitation, which has a heavy medical burden [4]. In addition, SAH is more common than other types of strokes, such as intracerebral hemorrhage and ischemic stroke, in young adults, which results in a more significant reduction in their productivity and a decline in their income [5]. The exact mechanism of brain injury caused by SAH remains unclear; thus, effective clinical treatments are lacking [6]. Therefore, it is important to explore the pathophysiological mechanisms of brain injury after SAH and develop targeted therapeutic strategies to improve the prognoses of SAH patients.

The pathophysiological mechanisms of brain injury post-SAH include the inflammatory response, blood‒brain barrier damage, neuronal death, oxidative stress, and autophagy [7], but the core pathological mechanism and the potential involvement of other mechanisms remain unclear. Recent studies have revealed the involvement of the immune response after SAH. Immune cells, including microglia and peripherally infiltrated neutrophils, are involved in brain injury post-SAH, and their inhibition alleviates brain injury [8, 9]. Peripheral monocytes and lymphocytes also infiltrate brain tissue after SAH [10], although whether other immune cell types are involved is unclear.

We performed bioinformatic analysis of multiomics data to explore the changes in the composition of immune cells after SAH. The immune cell types that exhibited the most significant and robust changes were identified and analyzed in vivo to determine their origin, infiltration mechanism, and role in brain injury after SAH.

## Materials and methods

### Experimental design

**Fig. 1:**
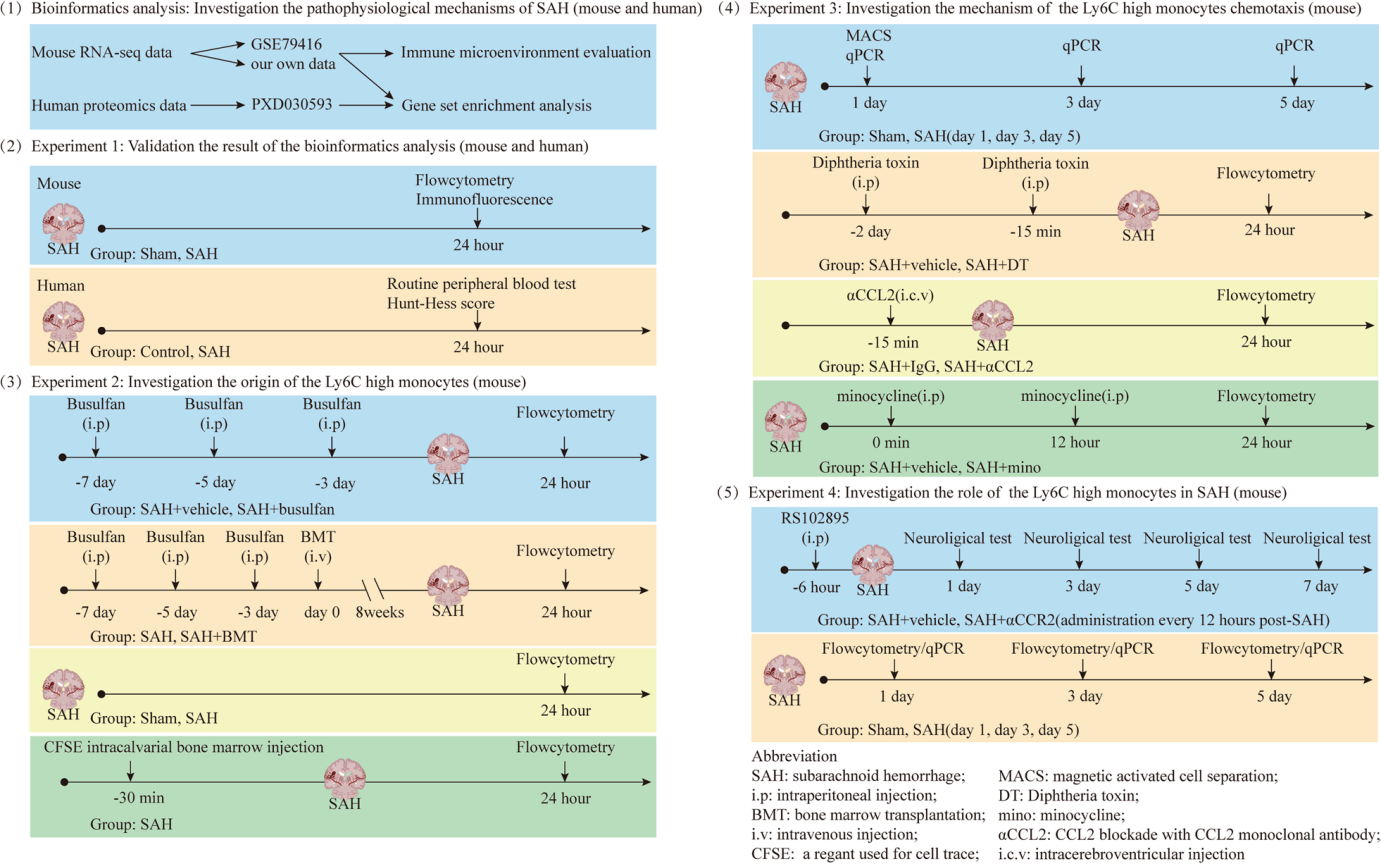
Experimental design and study groups

The study consisted of five parts, as depicted in Fig. 1.

1. Bioinformatics analysis: A total of 11 mice were used in this analysis, but 1 died; thus, data were obtained from 10 mice. To investigate the pathophysiological mechanisms of brain injury following SAH, bulk RNA-seq data from mice, including the published dataset GSE79416 and our own data (sham group, n = 6; SAH group, n = 4), and human proteomics data derived from a published dataset (PXD030593) were collected for gene set enrichment analysis (GSEA). Subsequently, based on the GSEA results, the immune microenvironment, particularly the dynamic changes in the immune cell composition, was assessed using two independent techniques: mMCP_counter and ImmuCellAI-mouse.
2. Experiment 1: A total of 19 mice were used in this experiment, and 2 of these mice died. To validate the findings of the bioinformatics analysis, we induced SAH in the mice (sham group, n = 8; SAH group, n = 9). We harvested the ipsilateral cerebral hemisphere of the mice for flow cytometry and immunofluorescence analyses to investigate the changes in Ly6C-high monocytes following SAH. Additionally, we collected routine peripheral blood test data from SAH patients (n = 365) and healthy controls (n = 347) to analyze the correlations between monocytes and the development of SAH.
3. Experiment 2: A total of 46 mice were used in this experiment, and 8 of the mice died. To ascertain whether Ly6C-high monocytes originated from the bone marrow, we depleted bone marrow cells through the administration of busulfan (SAH + vehicle group, n = 6; SAH + busulfan group, n = 6). Furthermore, we established chimeric mice with bone marrow cells expressing green fluorescent protein (GFP) to trace the origin (especially the cranial bone marrow and limb bone marrow) of Ly6C-high monocytes (SAH group, n = 2; SAH + BMT group, n = 8). Additionally, we examined the proliferative activity of Ly6C-high monocytes and their precursors, including monocyte-macrophage DC progenitors (MDPs) and common monocyte progenitors (cMoPs), in both the cranial bone marrow and limb bone marrow (sham group, n = 5; SAH group, n = 5). Subsequently, we used the cell tracer CFSE to further confirm the origin of Ly6C-high monocytes (SAH group, n = 6).
4. Experiment 3: A total of 77 mice were used in this experiment, and 12 of these mice died. To investigate the infiltration mechanism of Ly6C-high monocytes, we identified significantly upregulated chemokines by analyzing bulk RNA-seq data. These chemokines were then validated by qPCR experiments (sham group, n = 6; SAH group on day 1, n = 6; SAH group on day 3, n = 6; SAH group on day 5, n = 6). To determine the cell origin of the chemokines, we separated the brain cells into CD11b-positive cells (mainly microglia) and CD11b-negative cells and detected the expression of the identified chemokines by qPCR (sham group, n = 3; SAH group, n = 4). Subsequently, we assessed the percentage of Ly6C-high monocytes in the brain by inhibiting microglia with minocycline (mino) (SAH + vehicle group, n = 5; SAH + mino group, n = 5), specifically depleting microglia in Tmem119^DTR^ mice through the administration of diphtheria toxin (SAH + vehicle group, n = 6; SAH + DT group, n = 6), and using a CCL2 monoclonal antibody to antagonize CCL2 (αCCL2; SAH + IgG group, n = 6; SAH + αCCL2 group, n = 6).
5. Experiment 4: A total of 107 mice were used in this experiment, and 21 of the mice died. To elucidate the role of Ly6C-high monocytes in SAH, the mice were administered RS102895 to antagonize CCR2 (αCCR2), and their neurological function was assessed using the modified Garcia test, pole test, and rotarod test (SAH + vehicle group on days 1, 3, 5, and 7, n = 19, 18, 14, and 13, respectively; SAH + αCCR2 group on days 1, 3, 5, and 7, n = 19, 19, 14, and 11, respectively). Furthermore, to further investigate the mechanism by which Ly6C-high monocytes mediate neurological function recovery under SAH conditions, we evaluated the percentage and role of macrophages and monocyte-derived dendritic cells (moDCs) by flow cytometry (sham group, n = 6; SAH group on day 1, n = 6; SAH group on day 3, n = 18; SAH group on day 5, n = 12).

### Mice

C57BL/6J mice (male, weighing 22–27 g, less than 5 mice per cage, total of 260 mice) were purchased from SLAC Laboratory Animal (China) and maintained under constant temperature and humidity conditions with free access to food and water.

To antagonize the effect of CCR2, C57BL/6J mice were injected intraperitoneally with RS102895 (HY-18611, MedChemExpress, USA) at 5 mg/kg immediately and 6 h after surgery and then every 12 h until sacrifice [11].

To inhibit the activation of microglia, C57BL/6J mice were injected intraperitoneally with minocycline (mino, MS0037, Maokang Biotechnology, China) at 50 mg/kg immediately and 12 h after surgery [12].

Tmem119^DTR^ mice (male) expressing diphtheria toxin receptor (DTR) were established on a C57BL/6J background and purchased from Shanghai Model Organisms Center.

### Establishment of the SAH model

The endovascular perforation technique was used to establish the mouse model of SAH [13]. The mice were initially anesthetized with 5% isoflurane and maintained under 2% isoflurane throughout the experiment. After exposing the left carotid artery, the external and internal carotid arteries were carefully separated. The left external carotid artery (ECA) was isolated, and a blunt filament was advanced through the ECA into the internal carotid artery until resistance was encountered, which ultimately resulted in perforation of the circle of Willis. The sham mice were subjected to the same procedures except that the circle of Willis was not punctured. The mice were placed on a heating pad to maintain their body temperature during the surgery and afterward until they woke up. The severity of SAH was assessed based on the previously described SAH grading system [9] after the mice were sacrificed 24 h after the surgery (Additional file 1).

### RNA extraction and qPCR analysis

The mice were deeply anesthetized and transcardially perfused with cold 0.1 M phosphate-buffered saline (PBS), and the brain tissue was harvested. For mouse brain tissue RNA extraction, total RNA was isolated with TRIzol (AG21102, Accurate Biotechnology, Hunan, Co., Ltd). Total RNA from cells separated by magnetic activated cell separation (MACS) was isolated using an RNAprep Pure Micro Kit (DP420, Tiangen, China) according to the instructions.

cDNA was synthesized using Evo M-MLV RT Premix for qPCR (AG11706, Accurate Biotechnology, Hunan, Co., Ltd.). qPCR was performed with a SYBR Green Premix Pro Taq HS qPCR Kit (AG11701, Accurate Biotechnology, Hunan, Co., Ltd.) according to the manufacturer’s protocol. The relative mRNA expression levels of the target genes were normalized to that of β-actin using the 2^−ΔΔCt^ method. The forward and reverse primers of the genes are listed in Table 1.

**Table 1:** Sequences of the primers

Gene	Forward sequence (5′–3′)	Reverse sequence (5′–3′)
Actb	AGGCATTGTGATGGACTCCG	AGCTCAGTAACAGTCCGCCTA
Ccl2	CACTCACCTGCTGCTACTCA	GCTTGGTGACAAAAACTACAGC
Ccl3	TTCTCTGTACCATGACACTCTGC	CGTGGAATCTTCCGGCTGTAG
Ccl4	TTCCTGCTGTTTCTCTTACACCT	CTGTCTGCCTCTTTTGGTCAG
Ccl5	GCTGCTTTGCCTACCTCTCC	TCGAGTGACAAACACGACTGC
Ccl6	TGCCACACAGATCCCATGTA	GGTTCCCCTCCTGCTGATAA
Ccl7	CCCTGGGAAGCTGTTATCTTCAA	CTCGACCCACTTCTGATGGG
Ccl8	TCTACGCAGTGCTTCTTTGCC	AAGGGGGATCTTCAGCTTTAGTA
Ccl9	CCCTCTCCTTCCTCATTCTTACA	AGTCTTGAAAGCCCATGTGAAA
Itgax	CTGGATAGCCTTTCTTCTGCTG	GCACACTGTGTCCGAACTCA

### High-throughput RNA sequencing

The RNA amount and purity were quantified using a NanoDrop and Agilent 2100 bioanalyzer (Thermo Fisher, USA). The purified mRNA was fragmented into small pieces with fragment buffer at the appropriate temperature. First-strand cDNA was then generated by random hexamer-primed reverse transcription followed by second-strand cDNA synthesis. The cDNA fragments were then amplified by polymerase chain reaction, and the products were purified with AMPure XP Beads. The product was validated using an Agilent Technologies 2100 bioanalyzer for quality control. The double-stranded PCR products obtained in the previous step were heated, denatured and circularized based on the splint oligo sequence to obtain the final library. The final library was then amplified with phi29 to yield a DNA nanoball with more than 300 copies of one molecule. DNBs were loaded into the patterned nanoarray, and single-end 50-base reads were generated on the BGIseq500 platform (BGI-Shenzhen, China). The sequencing data were then filtered with SOAPnuke (v1.5.2), and clean reads were obtained and stored in FASTQ format. The following RNA-seq data analysis and visualization were performed in R (version 4.0.1) without special instructions.

### Gene set enrichment analysis (GSEA)

To determine the key biological processes that were altered after SAH, we performed a GSEA in the sham and SAH groups using our RNA-seq data and data downloaded from the Gene Expression Omnibus (GEO accession: GSE79416, mouse brain RNA-seq data, https://www.ncbi.nlm.nih.gov/geo/query/acc.cgi?acc=GSE79416) or ProteomeXchange (PXD030593, human cerebrospinal fluid proteomics data, https://proteomecentral.proteomexchange.org/cgi/GetDataset?ID=PXD030593) via GSEA software 4.0.1. Reference gene sets for Gene Ontology analysis (MousePath_GO_gmt.gmt) or (c5.go.bp.v7.5.1.symbols.gmt) were downloaded from the Gene Set Knowledgebase (http://www.ge-lab.org/gskb/) and Molecular Signatures Database (https://www.gsea-msigdb.org/gsea/msigdb/), respectively.

### Single-sample GSEA (ssGSEA)

The pathway enrichment score of each sample was calculated using the gsva function in the GSVA (gene set variation analysis) R package. “GO_BP_MM_POSITIVE_REGULATION_OF_MONOCYTE_CHEMOTAXIS” and “GO_BP_MM_MONOCYTE_CHEMOTAXIS” were downloaded from the Gene Set Knowledgebase (http://www.ge-lab.org/gskb/) to serve as reference gene sets.

### Evaluation of the immune microenvironment

Because immune cells are the major component of inflammation and immunity, we attempted to uncover the immunocyte composition based on bulk RNA-seq data using two independent techniques, mMCP_counter and ImmuCellAI-mouse [14, 15]. The results were normalized via the scale function in R to assess the consistency of the results between the two methods, and the interclass correlation coefficient (ICC) was calculated using the R package irr [16].

### Immunofluorescence staining

The mice were anesthetized by intraperitoneal injection of 1% pentobarbital sodium and transcardially perfused with cold 0.1 M PBS followed by cold 4% PFA (BL539A, Biosharp, China). After perfusion, the mouse brains were fixed in 4% PFA for 24 h and dehydrated in 30% sucrose until they sank to the bottom. The tissues were embedded in optimal cutting temperature compound and coronally sliced into 10-μm sections. The cryosections were blocked and permeabilized with blocking buffer (P0260, Beyotime, China, Cat. No.) for 1 h at room temperature. The sections were incubated with rabbit anti-CCR2 (ab273050, Abcam, UK) at 4 ℃ overnight and then with Alexa Fluor 488-conjugated secondary antibody (A-21206, Thermo Fisher, USA) for 1 h at room temperature. Subsequently, the sections were incubated with DAPI (ab104139, Abcam, UK), and Leica software was used for imaging.

### Bone marrow transplantation (BMT)

Male mice were injected intraperitoneally with 140 μL of 6 mg/mL busulfan (HY-B0245, MedChemExpress, USA) for the ablation of bone marrow cells at days -7, -5, and -3 before BMT. Donor bone marrow cells isolated from the hind limbs of GFP transgenic mice (R26-CAG-EGFP, male, kindly gifted by Dr. Wangwei Jing) were transferred into the recipient mice via vein injection on day 0 [17]. After 8 weeks under undisturbed conditions, the mice were used for the establishment of SAH model mice.

### Intracalvarial bone marrow injection

Intracalvarial bone marrow injection was conducted as described previously [18, 19]. Briefly, the mice were fixed using a stereotaxic apparatus, the occipital and parietal bones were slightly eroded by an electric drill, and 2 μL of CFSE solution (1:5 dilution, C34554, Thermo Fisher, USA) was manually injected using a 34G Hamilton syringe to trace the immunocytes in the skull bone marrow.

### Magnetic activated cell separation (MACS)

After transcardiac perfusion with 0.1 M PBS, the left hemisphere was harvested and then minced and ground to prepare single-cell suspensions. The cells were filtered through a 70-μm cell strainer (WHB-70UM, WHB Biotechnology, China) and subjected to 30% Percoll (17089109, Cytiva, USA) density gradient centrifugation to remove the myelin sheath. After lysing red blood cells with 1 × RBC LYSIS BUFFER SOLN (00-4333-57, Thermo Fisher, USA), cells were incubated with CD11b microbeads (130-093-636, Miltenyi Biotec, Germany) at 4 ℃ for 15 min and then applied onto MS columns (130-042-201, Miltenyi Biotec, Germany) to collect CD11b-positive and CD11b-negative cells.

### Microglia depletion

For microglia depletion, 80 ng of diphtheria toxin (DT) (0.5 ng/μL, 150, List Labs, USA) was injected intraperitoneally on day -2 and 0 before SAH induction.

### Intracerebroventricular injection (i.c.v)

CCL2 monoclonal antibody (16-7096-81, Thermo Fisher, USA) was used to antagonize the effect of CCL2. The mice were administered 4 μg/4 μL CCL2 monoclonal antibody or IgG control (16-4888-81, Thermo Fisher, USA) via i.c.v. injection as follows: the mice were anesthetized and fixed, a hole was drilled 0.2 mm posterior to the bregma and 1 mm lateral to the midline, and the injection was applied at a depth of 2.5 mm (1 μL/2 min). Before and after the i.c.v. injection, the needle was left in place for 5 and 10 min, respectively.

### Flow cytometry analysis

Single-cell suspensions were prepared as described above. After blocking the Fc receptors with anti-CD16/CD32 antibody and assessing the live or dead status of the cells using with Zombie NIR™ Fixable Viability Kit (423106, BioLegend, USA), the cells were incubated with cell surface antibodies (Table 2) for 30 min at 4 ℃. For detection of the intracellular TNF-α and IL-10 levels, the cells were incubated with a protein transport inhibitor cocktail (00-4980-93, Thermo Fisher, USA) for 6 h at 37 ℃ before cell surface antibody staining. After fixation and permeabilization with an Intracellular Fixation and Permeabilization Buffer Set (88-8824-00, Thermo Fisher, USA), the cells were harvested and incubated with TNF-α (11-7321-81, Thermo Fisher, USA) and IL-10 (564083, Becton, Dickinson, and Company, USA) antibodies. The proliferative activity of Ly6C-high monocytes and their progenitor cells (MDPs and cMoPs) in the bone marrow was detected by Ki-67 staining. The two-chamber method was used to harvest a single-cell suspension of the hind limb bone marrow [20]; the cranium was cut into pieces and centrifuged at 500×*g* for 5 min to obtain a single-cell suspension. The single-cell suspensions were then filtered, stained with cell surface antibody, fixed, permeabilized with the Foxp3/Transcription Factor Staining Buffer Set (00-5523-00, Thermo Fisher, USA), and then stained with Ki-67 antibody (11-5698-82, Thermo Fisher, USA).

**Table 2:** Cell surface antibody used in flow cytometry analysis

Reagent	Source	Identifier
CD45	Thermo Fisher	11-0454-85
CD45	Thermo Fisher	56-0454-82
CD11b	BioLegend	101212
CD11b	Thermo Fisher	25-0112-82
Ly6G	BioLegend	127616
Ly6G	BioLegend	127608
Ly6G	Thermo Fisher	63-9668-82
Ly6C	BioLegend	128033
F4/80	Thermo Fisher	48-4801-82
CD11c	BioLegend	117308
B220	Thermo Fisher	56-0452-82
CD64	BioLegend	139313
XCR1	BioLegend	148207
CD172a	BioLegend	144013
CD115	BioLegend	135505
c-kit	BioLegend	105841

### Evaluation of neurological function

The Modified Garcia test includes spontaneous activity, movement symmetry of the four limbs, forelimb outstretching, climbing, body proprioception, and response to vibrissae touch, and a higher score represents better neurological performance [21]. Before modeling, the mice were subjected to pole and rotarod tests three times a day (3 days in total), and the results from the last test were recorded as the baselines. For the pole test [22], the time of the 180° turn was recorded as Tturn, and the time to descend to the bottom was recorded as Ttotal. If Tturn > 30 s, Tturn and Ttotal were both recorded as 30 s, and if the mice successfully turned within 30 s but fell or slipped or the Ttotal > 30 s, the Ttotal was recorded as 30 s. For the rotarod test [23], the initial speed of the rotating rod was 5 rpm, the acceleration was 0.2 rpm/s, and the maximum speed was 65 rpm. When the mice fell, the time and speed were recorded. The neurological function evaluation was performed by a researcher blinded to the experimental design.

### Collection of clinical data

Routine peripheral blood data of patients with aneurysmal SAH and individuals at the physical examination center (as healthy controls) were collected between May 2018 and June 2020 at the hospital. For the patients, routine peripheral blood tests were performed within 24 h after SAH. The data from patients with a history of previous stroke, infectious diseases, inflammatory diseases, or tumors, using immunosuppressive agents, and with liver, kidney, or heart dysfunction were excluded. Using the case‒control matching function in SPSS software, the patients in the SAH and healthy control groups were matched based on clinical characteristics, including age, sex, hypertension, and diabetes (Table 3).

**Table 3:** Clinical characteristics of the healthy controls and SAH patients

	Healthy controls (n = 347)	SAH patients (n = 365)	p value
Age	55.52 ± 10.72	56.46 ± 11.71	0.3063
Female	212 (61.10)	230 (63.01)	0.5979
Hypertension	159 (45.82)	176 (48.21)	0.5217
Diabetes	18 (5.19)	18 (4.93)	0.8763

The age is presented as the mean ± SD and tested using the Mann‒Whitney test, and the other variables, namely, female sex, hypertension and diabetes, are presented as n (%) values and tested using Chi-square tests

### Statistical analysis

The normality and variance homogeneity of the data were analyzed based on the Shapiro‒Wilk and Levene methods, respectively. If the data met the assumptions of normality and homogeneity of variance, the significance of the data was tested by Student’s t test (two groups) or one-way analysis of variance (ANOVA) (multigroup) followed by Tukey’s post hoc test. If the data were nonnormally distributed, they were tested using the Mann–Whitney test (two groups) and Kruskal‒Wallis test (multigroup) followed by Dunn’s post hoc test. The results from the neurological function tests were analyzed by two-way ANOVA followed by the Sidak post hoc test. Typically, P values lower than 0.05 are considered to indicate significance. All statistical analyses were performed with SPSS 26 and GraphPad Prism 8.

## Results

### The monocyte levels increase after SAH

**Fig. 2:**
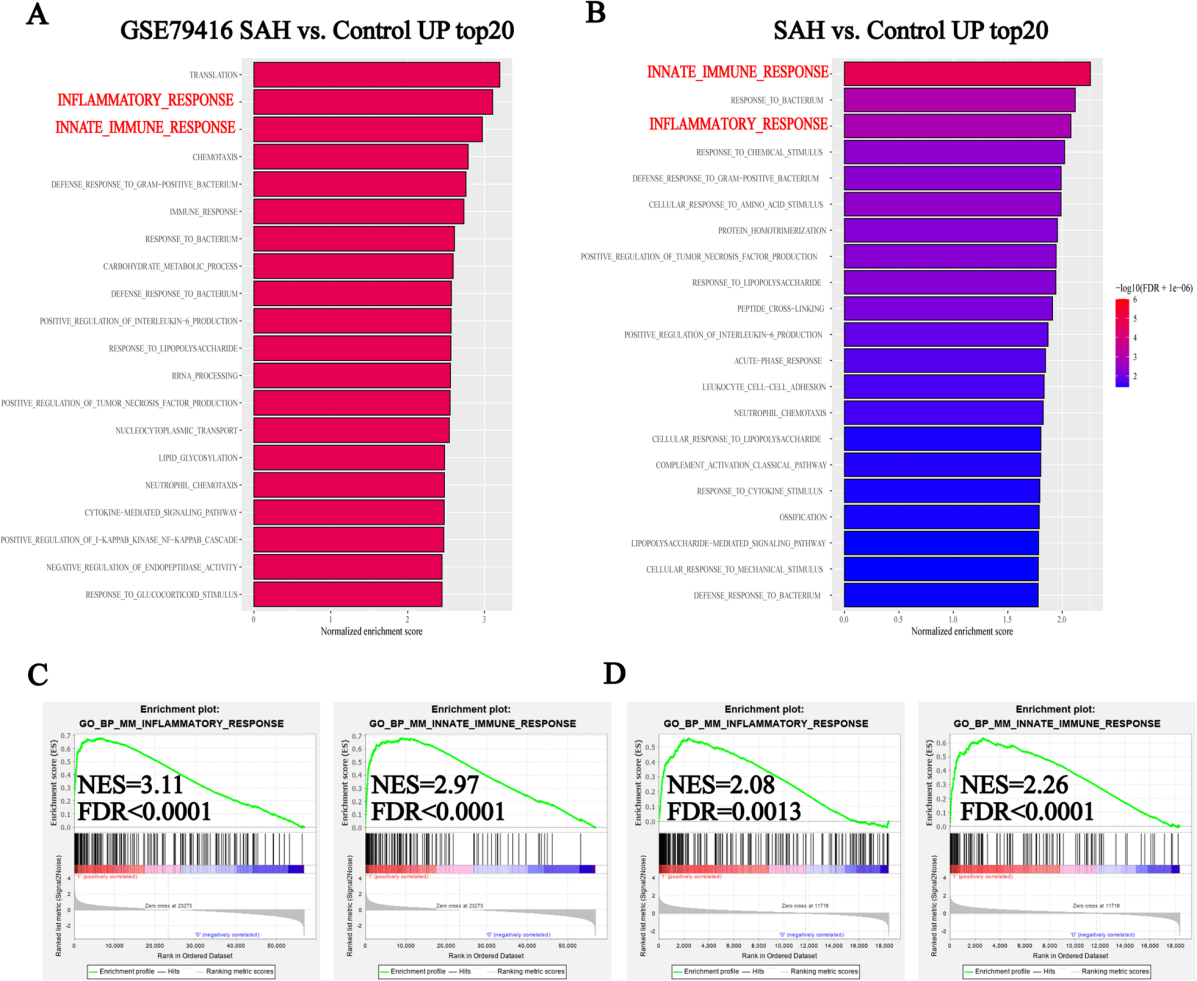
Results from the GSEA of GSE79416 (**A**) and our data (**B**) showing the top 20 upregulated biological processes after SAH. Plots showing the enrichment of the inflammatory response and immune response pathways in GSE79416 (**C**) and our data (**D**) (GSE79416, n = 3/group; sham group (our data), n = 6; SAH group (our data), n = 4)

**Fig. 3:**
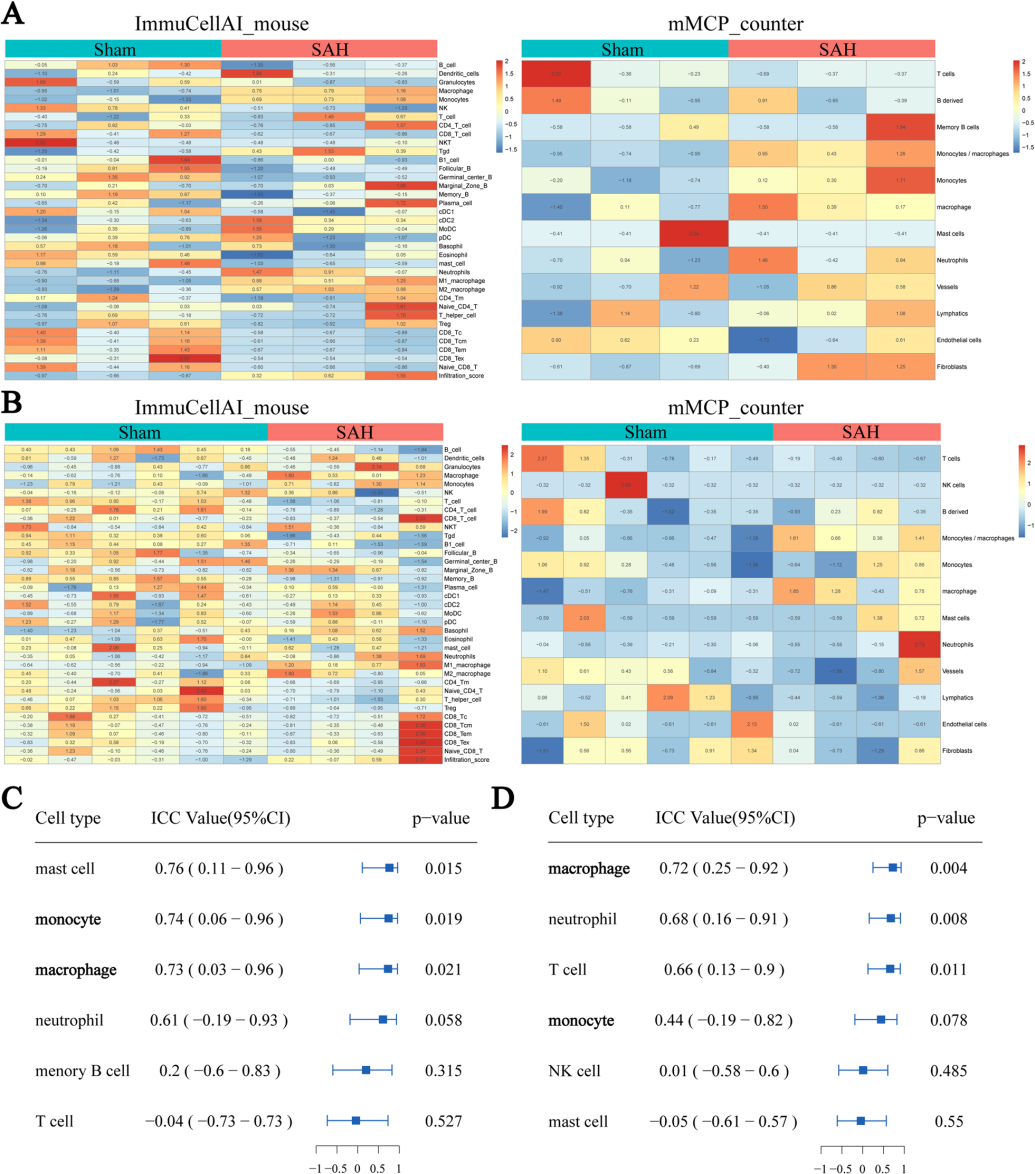
**A**, **B** Heatmap plots showing the constitution of immunocytes in GSE79416 (**A**) and our data (**B**) calculated using ImmuCellAI_mouse and mMCP_counter (GSE79416, n = 3/group; sham group (our data), n = 6; SAH group (our data), n = 4). **C**, **D** Forest plots showing the ICC values between ImmuCellAI_mouse and mMCP_counter obtained for GSE79416 (**C**) and our data (**D**) using the ICC algorithm

**Fig. 4:**
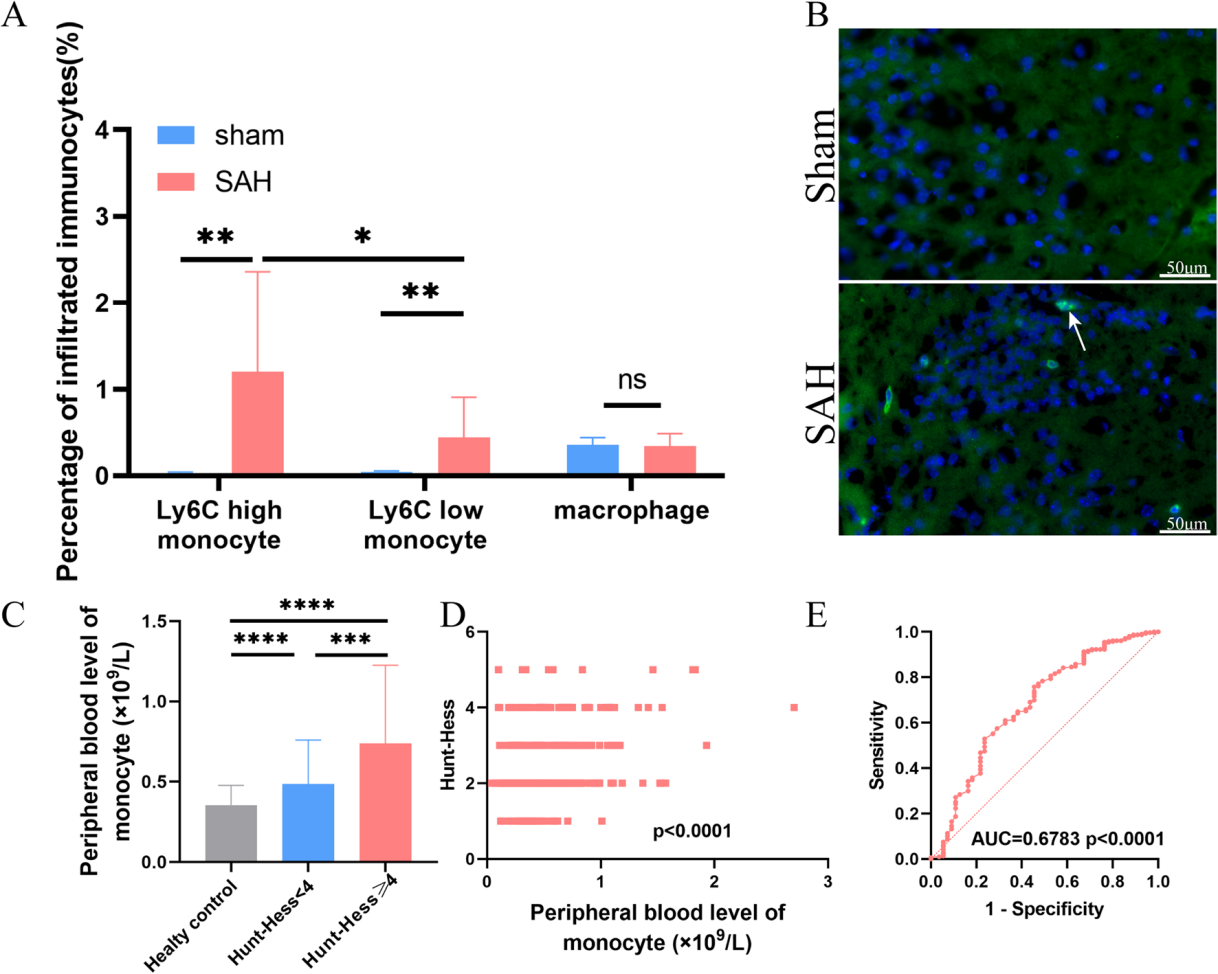
**A** Percentage of Ly6C-high monocytes, Ly6C-low monocytes, and macrophages in mouse brains determined by flow cytometry on day 1 post-surgery (sham group, n = 5; SAH group, n = 6). **B** CCR2 (green)-positive cells (white arrow) in the ipsilateral basal cortex of the mice were assessed by immunofluorescence staining on day 1 post-surgery (n = 3/group). **C** Distribution of monocytes in the peripheral blood of healthy controls, SAH patients with Hunt-Hess scores < 4 and SAH patients with Hunt-Hess scores ≥ 4 (healthy controls, n = 347; Hunt-Hess < 4, n = 310; Hunt-Hess ≥ 4, n = 55). **D** ROC curve showing the discriminating value of monocytes in the peripheral blood between SAH patients with Hunt-Hess scores < 4 and SAH patients with Hunt-Hess scores ≥ 4 (Hunt-Hess < 4, n = 310; Hunt-Hess ≥ 4, n = 55). **E** The correlation between peripheral blood monocytes and the Hunt-Hess score (n = 365). ns, P ≥ 0.05; *P < 0.05; **P < 0.01; ***P < 0.001; ****P < 0.0001

The results from a GSEA of two independent RNA-seq datasets (Additional file 2) showed that the inflammatory response and immune response, especially the innate immune response, was significantly enriched in the SAH group compared to the control group (Fig. 2). Similarly, proteomics data from the cerebrospinal fluid of patients revealed the activation of innate immunity and acute inflammatory response pathways (Additional file 3). The levels of several immunocytes, including macrophages and monocytes, in the mouse brain tissues changed after SAH; however, some discrepancy in the cell type trends, e.g., T cells, was found between the mMCP_counter and ImmuCellAI-mouse techniques (Fig. 3A, B; Additional file 4). The ICC for the common cell types demonstrated that the levels of monocytes and macrophages were consistent between the two RNA-seq datasets, indicating that the counts of monocytes and macrophages robustly increased after SAH (Fig. 3C, D). This finding was corroborated by a flow cytometry analysis of brain tissue, which revealed a significant increase in the levels of monocytes, particularly Ly6C-high monocytes; however, little change in the macrophage density was observed (Fig. 4A; Additional file 5). Immunofluorescence demonstrated an increase in CCR2-positive cell infiltration into mouse brain tissue after SAH (Fig. 4B). Consistently, the results from routine peripheral blood tests revealed significantly higher monocyte concentrations in SAH patients than healthy individuals, and these concentrations were positively correlated with the Hunt-Hess scores (Fig. 4C, D) and could be used to distinguish between SAH patients with Hunt-Hess scores ≥ 4 and those with Hunt-Hess scores < 4 (Fig. 4E). This finding suggests a positive correlation between monocytes and SAH disease severity.

### Ly6C-high monocytes derived from bone marrow

**Fig. 5:**
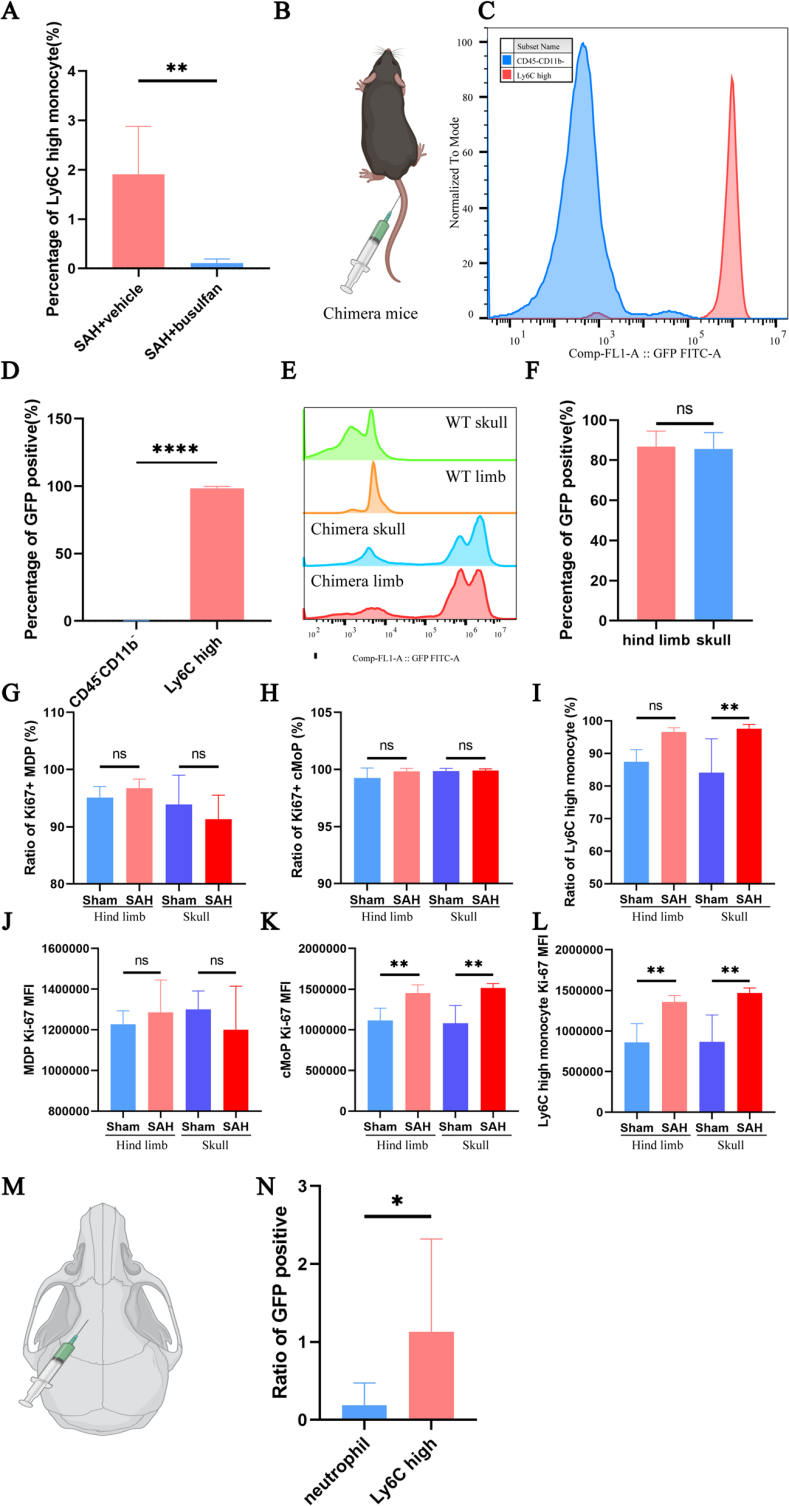
**A** The percentage of Ly6C-high monocytes on day 1 post-surgery was assessed by flow cytometry (n = 6/group). **B** Schematic diagram of the establishment of chimeric mice. **C**, **D** The percentage of GFP-positive Ly6C-high monocytes on day 1 post-surgery was assessed by flow cytometry (n = 6). **E**, **F** The percentage of GFP-positive cells in the hind limb and calvaria of the wild-type (WT) and chimeric mice on day 1 post-surgery was assessed by flow cytometry (n = 2). **G**–**L** The percentage of Ki-67-positive and associated mean fluorescence intensity (MFI) of MDPs, cMoPs, and Ly6C-high monocytes on day 1 post-surgery was assessed by flow cytometry (n = 5/group). **M**, **N** Schematic diagram of intracalvarial bone marrow injection and GFP-positive ratios in neutrophils and Ly6C-high monocytes on day 1 post-surgery was assessed by flow cytometry. GFP-positive ratio = brain GFP-positive ratio/skull GFP-positive ratio, n = 6. ns, P ≥ 0.05; *P < 0.05; **P < 0.01; ***P < 0.001; ****P < 0.0001

A significant decrease in the proportion of infiltrated Ly6C-high monocytes was observed after the depletion of bone marrow cells (Fig. 5A). Consistently, the majority of Ly6C-high monocytes in the brains of bone marrow chimeric mice were positive for GFP (Fig. 5B–D). The GFP ratio obtained for the calvarial and hind limb bone marrow was equivalent to that found for the chimeric mice (Fig. 5E, F), and cMoPs and Ly6C-high monocytes exhibited increased proliferative activity after SAH (Fig. 5G, L; Additional file 6). Ly6C-high monocytes showed fluorescence after injection of a cell tracer (CFSE) into the calvarial bone marrow, and the contribution of calvarial bone marrow to Ly6C-high monocytes was higher than that to neutrophils (Fig. 5M, N).

### Microglia drive Ly6C-high monocyte chemotaxis

**Fig. 6:**
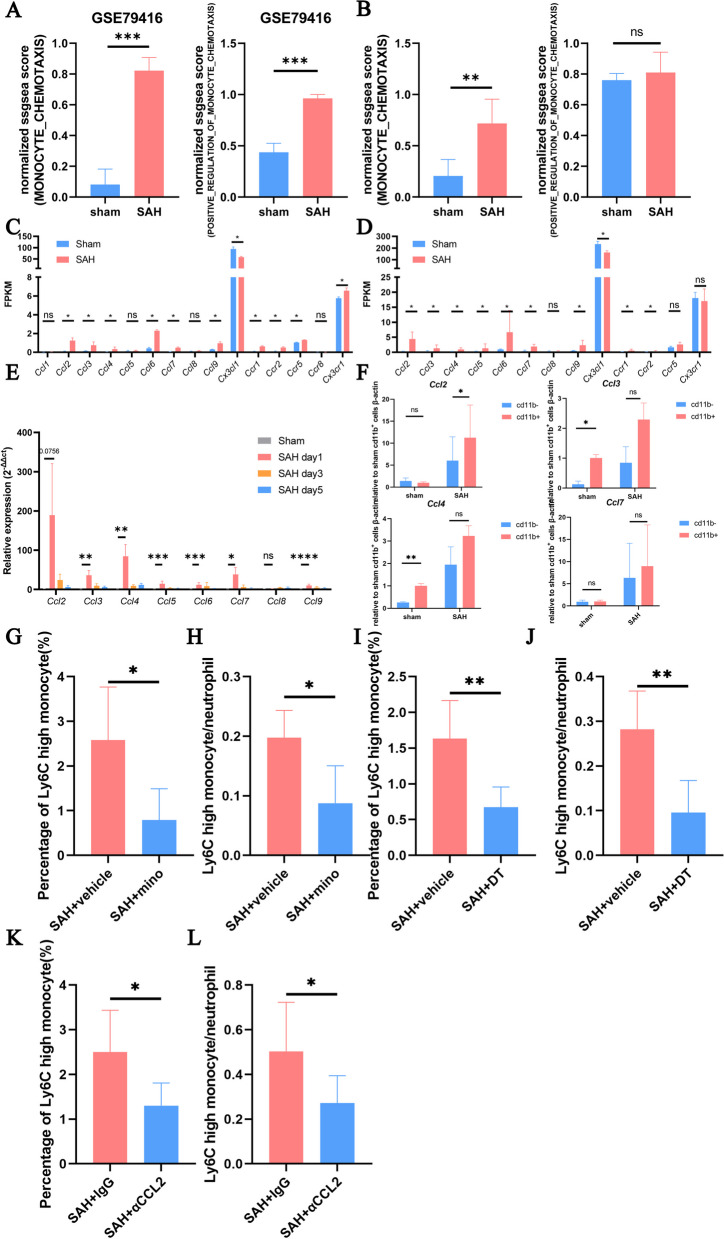
**A**, **B** ssGSEA scores of the MONOCYTE_CHEMOTAXIS and POSITIVE_REGULATION_OF_MONOCYTE_CHEMOTAXIS pathways in GSE79416 and our data. **C**, **D** FPKM values of Ccl1, Ccl2, Ccl3, Ccl4, Ccl5, Ccl6, Ccl7, Ccl8, Ccl9, Cx3cl1, Ccr1, Ccr2, Ccr8, and Cx3cr1 in GSE79416 and our data. **E** The mRNA expression levels of Ccl2, Ccl3, Ccl4, Ccl5, Ccl6, Ccl7, Ccl8, and Ccl9 in the mouse brains on day 1 post-surgery were assessed by qPCR (n = 6/group). **F** The mRNA expression levels of Ccl2, Ccl3, Ccl4, and Ccl7 in CD11b-positive and -negative cells isolated from sham and SAH mouse brains on day 1 post-surgery were assessed by MACS (sham group, n = 3; SAH group, n = 4). **G**, **H** The percentage of Ly6C-high monocytes and the ratio of Ly6C-high monocytes to neutrophils in the brains of mice in the SAH + vehicle and SAH + mino groups on day 1 post-surgery were assessed by flow cytometry (n = 5/group). **I**, **J** The percentage of Ly6C-high monocytes and the ratio of Ly6C-high monocytes to neutrophils in the brains of mice in the SAH + vehicle and SAH + DT groups on day 1 post-surgery were assessed by flow cytometry (n = 6/group). **K**, **L** The percentage of Ly6C-high monocytes and the ratio of Ly6C-high monocytes to neutrophils in the brains of mice in the SAH + IgG and SAH + αCCL2 groups on day 1 post-surgery were assessed by flow cytometry (n = 6/group). ns, P ≥ 0.05, *P < 0.05; **P < 0.01; ***P < 0.001; ****P < 0.0001

Pathways including monocyte chemotaxis and positive regulation of monocyte chemotaxis were found to be activated after SAH (Fig. 6A, B). Many monocyte-related chemokines and receptors were upregulated after SAH (Fig. 6C, D), and the expression of Ccl2, Ccl3, Ccl4, Ccl5, Ccl6, Ccl7, and Ccl9 was confirmed by qPCR (Fig. 6E), which revealed that Ccl2 was predominantly expressed on CD11b-positive cells (Fig. 6F). The percentage of Ly6C-high monocytes and the ratio of Ly6C-high monocytes to neutrophils decreased after mino administration (Fig. 6G, H). The depletion of microglia in Tmem119^DTR^ mice with DT and the blockade of CCL2 yielded similar results (Fig. 6I–L).

### Ly6C-high monocytes contribute to neurological function recovery after SAH

**Fig. 7:**
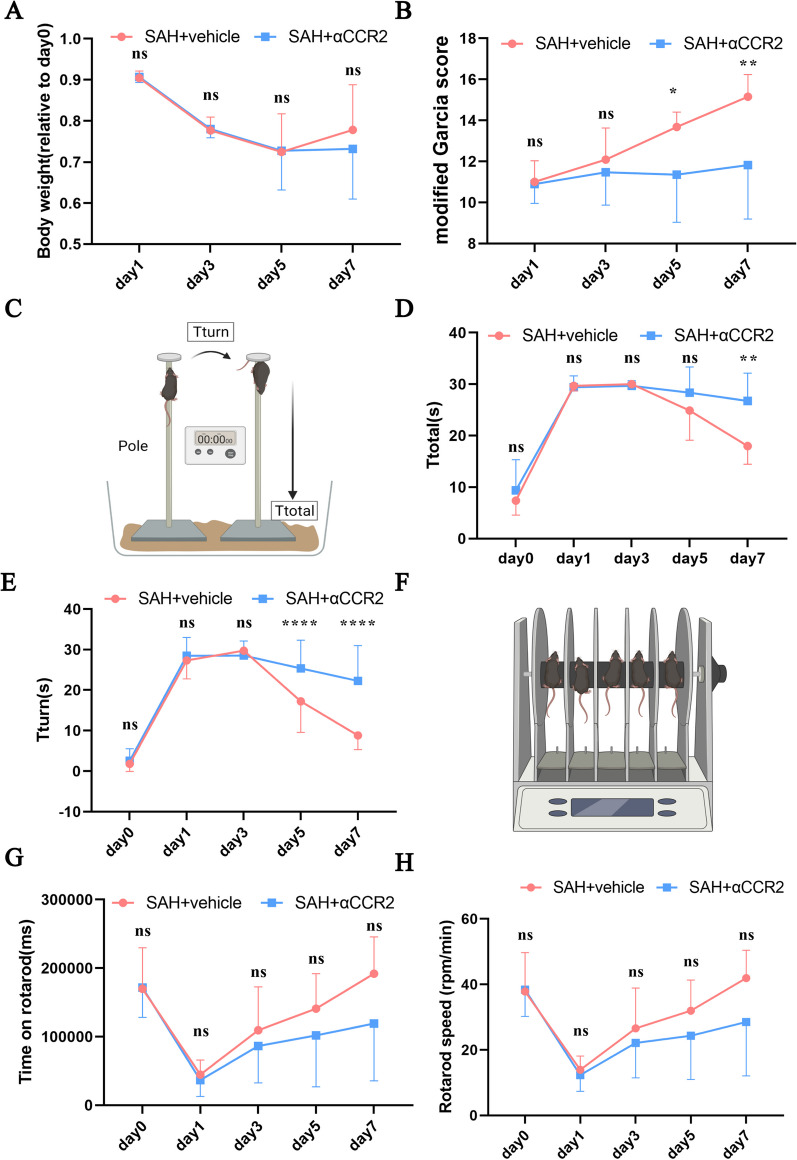
**A** Change in the body weight of the SAH + vehicle and SAH + αCCR2 mice (SAH + vehicle group on day 1, 3, 5, and 7, n = 19, 18, 14, and 13, respectively; SAH + αCCR2 group on day 1, 3, 5, and 7, n = 19, 19, 14, and 11, respectively). **B** Modified Garcia score of the SAH + vehicle and SAH + αCCR2 mice (SAH + vehicle group on day 1, 3, 5, and 7, n = 19, 18, 14, and 13, respectively; SAH + αCCR2 group on day 1, 3, 5, and 7, n = 19, 19, 14, and 11, respectively). **C** Schematic diagram of the pole test. **D**, **E** Ttotal and Tturn of the SAH + vehicle and SAH + αCCR2 mice (SAH + vehicle group on day 1, 3, 5, and 7, n = 19, 18, 14, and 13, respectively; SAH + αCCR2 group on day 1, 3, 5, and 7, n = 19, 19, 14, and 11, respectively). **F** Schematic diagram of the rotarod test. **G**, **H** Time on the rotarod and speed at which the SAH + vehicle and SAH + αCCR2 mice fell off (SAH + vehicle group on day 1, 3, 5, and 7, n = 19, 18, 14, and 13, respectively; SAH + αCCR2 group on day 1, 3, 5, and 7, n = 19, 19, 14, and 11, respectively). ns, P ≥ 0.05, *P < 0.05; **P < 0.01; ***P < 0.001; ****P < 0.0001

Weight recovery appeared to be slower in the SAH + αCCR2 group than in the SAH + vehicle group, although the difference was not significant (Fig. 7A). After the evaluation of neurological function, the mice in the SAH + αCCR2 group showed a slower increase in the modified Garcia score, slower decreases in Tturn and Ttotal, and a slower increase in the time on the rotarod compared with the mice in the SAH + vehicle group (Fig. 7B–H).

### Differentiation of Ly6C-high monocytes into moDCs and macrophages

**Fig. 8:**
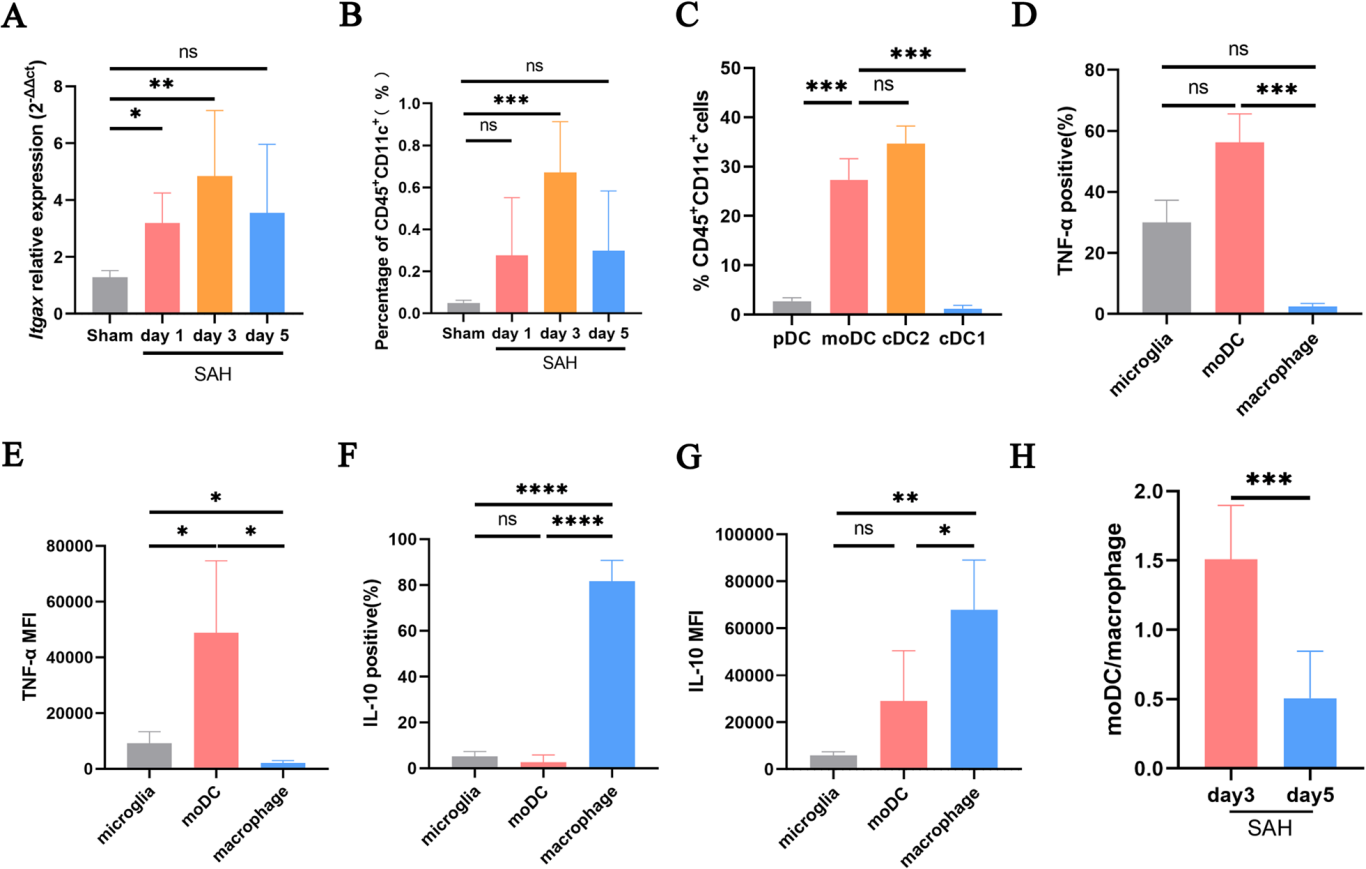
**A** mRNA level of Itgax in the mouse brains (n = 6/group). **B** The percentage of CD45^+^CD11c^+^ cells in the mouse brains was detected via flow cytometry (n = 6/group). **C** The proportions of pDC, cDC1, cDC2 and moDCs in CD45^+^CD11c^+^ cells in the mouse brains on day 3 post-SAH were assessed via flow cytometry (n = 6). **D**, **E** The percentage of TNF-α-positive and associated MFI in the mouse brains on day 3 post-SAH was assessed by flow cytometry (n = 6). **F**, **G** The percentage of IL-10-positive and associated MFI in the mouse brains on day 3 post-SAH was assessed by flow cytometry (n = 6). **H** Ratio of moDCs to macrophages on day 3 and 5 after SAH (n = 6/group). ns, P ≥ 0.05, *P < 0.05; **P < 0.01; ***P < 0.001; ****P < 0.0001

Previous studies confirmed that Ly6C-high monocytes differentiated into moDCs and macrophages [24]. Because few studies have investigated moDCs, we first focused on moDCs. Dendritic cells (DCs) can be classified into four lineages: conventional dendritic cells (cDCs, including cDC1 and cDC2), plasmacytoid DCs (pDCs) and moDCs. Thus, moDCs also express the DC marker gene Itgax, which is the gene encoding CD11c. The mRNA level of Itgax increased at 1 day and peaked at 3 days after SAH (Fig. 8A), and similar trends were found for CD45^+^CD11c^+^ cells, as revealed by flow cytometry (Fig. 8B). Subsequent analysis revealed that moDCs were the second-largest DC subpopulation (Fig. 8C; Additional file 7). Additionally, the moDC population exhibited a higher proportion of TNF-α-positive cells and a lower proportion of IL-10-positive cells than the macrophage population (Fig. 8D–G). The ratio of moDCs to macrophages was higher on day 3 than on day 5 after SAH (Fig. 8H).

## Discussion

The mechanisms of brain injury after SAH are complex and poorly elucidated but needed for the development of drugs that can improve SAH patient prognoses [9]. The bioinformatic analysis indicated predominant roles of the inflammatory and immune responses after SAH, as revealed by a significant increase in mouse Ly6C-high monocytes in mouse models. Consistently, routine peripheral blood data from SAH patients showed increased monocyte counts, as previously documented [25], indicating that Ly6C-high monocytes may be important during brain injury after SAH.

Although monocytes likely originate from the spleen or bone marrow [26, 27], the origin of Ly6C-high monocytes in SAH has not yet been confirmed. Bone marrow chimeric mice confirmed that the origin of Ly6C-high monocytes produced after SAH was the bone marrow. Recent studies showed that the limb bone marrow and cranial bone marrow produce immune cells involved in central nervous system (CNS) disease [18, 19]. Additionally, Ly6C-high monocytes that infiltrate the CNS are more dependent on the cranial bone marrow than neutrophils [18, 19], corroborating our findings, potentially because neutrophils are more prolific in the peripheral blood or bone marrow.

Chemokines associated with monocyte chemotaxis, especially Ccl2, were significantly upregulated after SAH, as previously detected in the cerebrospinal fluid and peripheral blood [28, 29]. The CCL2-activated receptor CCR2 expressed on Ly6C-high monocytes contributes to their migration into the brain [30], as confirmed by the finding that the blockade of CCL2 led to reduced Ly6C-high monocyte infiltration, highlighting its chemotactic effect [31]. Although the cellular origin of CCL2 has been proposed to be secretion from a variety of cells, even neurons, it remains unclear [32]. We found that CCL2 was predominantly upregulated in CD11b-positive cells, mainly microglia, and that the depletion or inhibition of microglia reduced Ly6C-high monocyte infiltration, suggesting a key role of microglia in mediating Ly6C-high monocyte infiltration after SAH.

The inhibition of Ly6C-high monocytes slowed neurological recovery in the subacute phase (days 5–7) after SAH. Ly6C-high monocytes may therefore play a protective role in SAH, as in other stroke types in which the knockdown of CCR2 improves the neurological function of mice [30, 33, 34]. Monocytes can differentiate into moDCs and macrophages [35], and fate mapping experiments have confirmed that moDCs are derived from Ly6C-high monocytes [36]. moDCs have been found in a variety of acute and chronic CNS diseases, including stroke and experimental autoimmune encephalomyelitis (EAE) [37, 38], and might play a potentially pathogenic role in mediating inflammation and demyelination [38, 39]. Consistently, we found that moDCs were more proinflammatory and exhibited a higher TNF-α-producing capacity than macrophages. We observed that the balance of Ly6C-high monocyte differentiation gradually shifted away from moDCs toward macrophages as the acute phase transitioned to the subacute phase. We speculate that cells that differentiated from Ly6C-high monocytes play various roles at different stages after SAH. The results indicate that Ly6C-high monocytes and moDCs play a proinflammatory role in the acute phase and that macrophages play an anti-inflammatory and reparative role in the subacute phase of SAH. Regarding the specific molecular mechanism by which macrophages promote SAH recovery, bulk RNA-seq data from monocytes/macrophages after intracerebral hemorrhage might provide some hints. In the acute stage, upregulation of proinflammatory genes, including cytokines, chemokines, and interferon-related genes, such as Ifnar2, Mif, Il1a, Il1b, Tnf, Il6st, Ccl2, Ccl3, Ccl4, Ccl9, Ccr5, Isg20, Isg15, Ifit2, Ifit3, Irf7, Rsad2, Bst2, Stat1, Sell, Rel, Cxcl10, Ccrl2, Ddx58 (RIG-I), and Fcgr1, has been observed. Conversely, upregulation of anti-inflammatory and reparative genes, including Axl (associated with efferocytosis and cytokine inhibition), Lamp1 (related to phagolysosome formation), Cd63 (involved in phagolysosome formation), Abcg1 (associated with lipid efflux), and Anxa1 (contributing to inflammation resolution), has been detected in the subacute stage [33]. However, the specific molecular mechanism still needs further study.

In summary, our data provide new insights into the origin, infiltration mechanism, and role of Ly6C-high monocytes in brain injury after SAH. More importantly, both our study and the published literature indicate that Ly6C-high monocytes play multiple roles after stroke and thus contribute to not only proinflammatory activities but also reparative processes. These insights compel a re-evaluation of the conventional understanding of Ly6C-high monocyte function during disease progression and advocate for a more nuanced strategy in which interventions are meticulously tailored based on the temporal role of Ly6C-high monocytes during different disease phases. Instead of suppressing these monocytes throughout the whole course of the disease, a more targeted approach, such as promoting their differentiation into reparative macrophages during the chronic phase, might be more beneficial for patient recovery. Furthermore, recent evidence suggests a paradoxical effect of anti-inflammatory drugs, including steroid or nonsteroidal anti-inflammatory drugs (NSAIDs). Although they provide short-term analgesic relief in the acute phase, these drugs might also provoke the transition from acute to chronic pain, potentially due to their dampening effect on the acute inflammatory response [40]. This body of research, including our own, proposes a complex interplay between the suppression of acute inflammation and the subsequent implications for disease resolution, which underscores the notion that while dampening inflammation might be advantageous in the acute stages of the disease, it could inadvertently delay the healing process. Therefore, more studies are needed to unravel the intricate relationship between the inflammatory response and tissue repair and to guide more effective and targeted therapeutic strategies.

Our study also has various limitations. (1) Sex is an essential factor in the pathological processes and outcomes of SAH. Further work to confirm the origin, infiltration mechanism, and role of monocytes in a female SAH mouse model is needed. (2) The present work focused on confirming the role of Ly6C-high monocytes in SAH, and future studies will attempt to explore the role of moDCs and macrophages in SAH. (3) In addition to pharmacological inhibition of CCR2, it is also necessary to introduce CCR2 knockout mice to confirm the results. (4) The underlying mechanism by which Ly6C-high monocytes promote neurological function recovery still needs further study.

## Conclusion

**Fig. 9:**
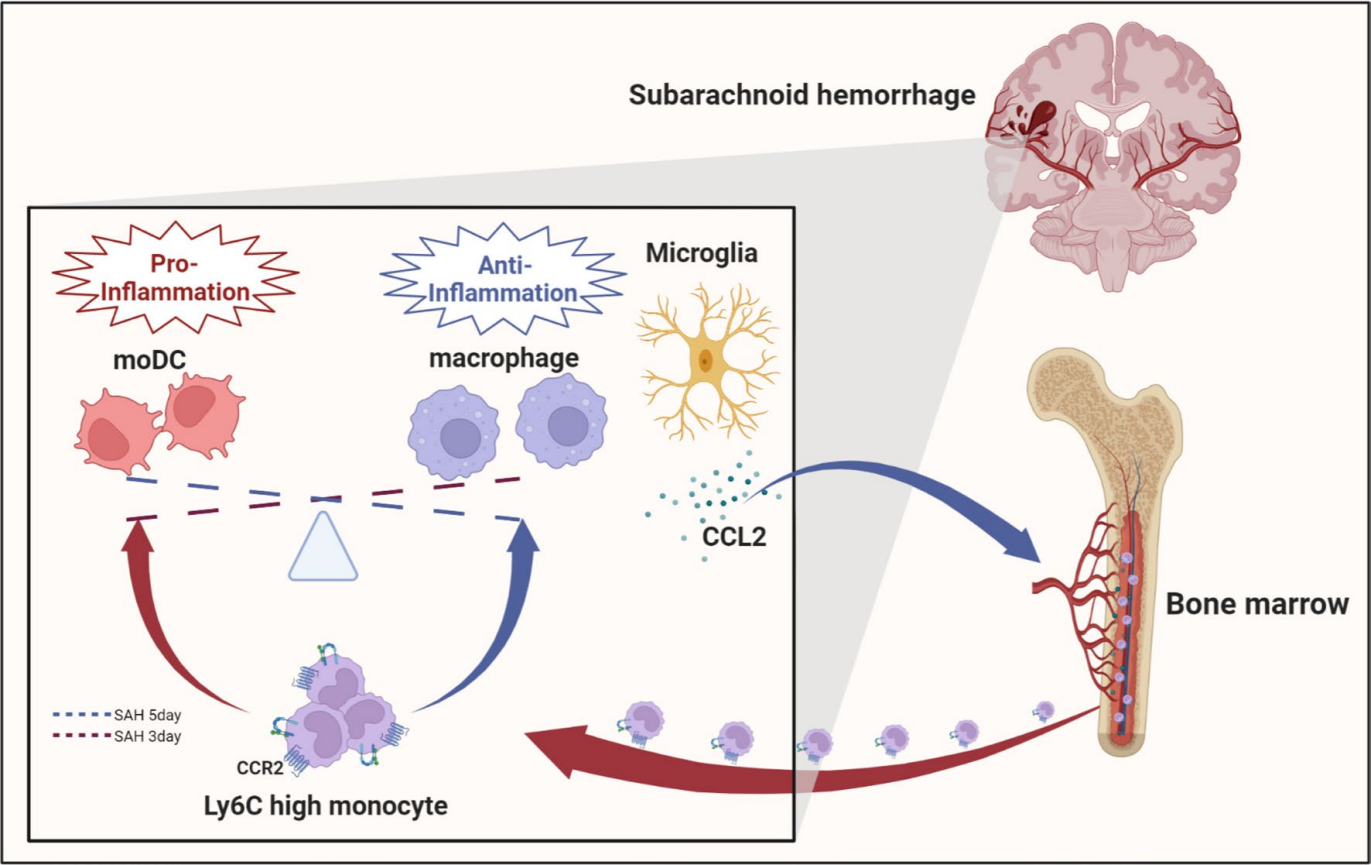
Schematic diagram of the role and mechanism of Ly6C-high monocytes in SAH

We found that inflammatory and immune responses were the core pathological mechanisms of brain injury caused by SAH. Monocytes, especially Ly6C-high monocytes, showed robust and significant increases in number after SAH and infiltrated the brain from the skull and limb bone marrow through a process driven by CCL2 secreted by microglia. Ly6C-high monocytes promoted the recovery of neurological function after SAH (Fig. 9).

### Supplementary Information

**Additional file 1:** (A) Study groups and number of mice in each group. (B) Representative images of brains harvested from the sham and SAH mice. (C) SAH grade of each group in the study.

**Additional file 2:** RNA-seq data obtained in this study.

**Additional file 3:** (A) Volcano plots showing upregulated and downregulated pathways in PXD030593. The upregulated biological processes were defined as those with NES > 1, p value < 0.05, and FDR < 0.25, whereas the downregulated biological processes were defined as those with NES < -1, p value < 0.05, and FDR < 0.25. (B) Plots showing the enriched pathways including activation of the immune response, positive regulation of the immune response, innate immune response, and acute inflammatory response in PXD030593.

**Additional file 4:** Bar plots showing the constitution of immunocytes in GSE79416 (A) and our data (B) calculated using ImmuCellAI_mouse and mMCP_counter.

**Additional file 5:** Gating strategy for Ly6C-high monocytes (CD45^high^CD11b^+^Ly6G^−^Ly6C^high^F4/80^−^), Ly6C-low monocytes (CD45^high^CD11b^+^Ly6G^−^Ly6C^low^F4/80^−^), and macrophages (CD45^high^CD11b^+^Ly6G^−^F4/80^+^).

**Additional file 6:** Gating strategy for MDPs (R1, CD115^+^CD135^+^c-kit^+^Ly6C^−^CD11b^−^), cMoPs (R2, CD115^+^CD135^−^c-kit^+^Ly6C^+^CD11b^−^), and Ly6C-high monocytes (R3, CD115^+^CD135^−^c-kit^−^Ly6C^+^CD11b^+^) in the bone marrow.

**Additional file 7:** Gating strategy for pDCs (CD45^high^CD11c^+^CD45R^+^), cDC1 (CD45^high^CD11c^+^CD45R^−^Ly6C^−^CD64^−^XCR1^+^CD172A^−^), cDC2 (CD45^high^CD11c^+^CD45R^−^Ly6C^−^CD64^−^XCR1^−^CD172A^+^), and moDCs (CD45^high^CD11c^+^CD45R^−^CD64^+^).

## Data Availability

The datasets used and/or analyzed during the current study are available from the corresponding author upon reasonable request.

## Abbreviations

SAH: Subarachnoid hemorrhage
mino: Minocycline
αCCL2: Antagonize CCL2
αCCR2: Antagonize CCR2
moDCs: Monocyte-derived dendritic cells
GSEA: Gene set enrichment analysis
GFP: Green fluorescent protein
MDPs: Monocyte-macrophage DC progenitors
cMoPs: Common monocyte progenitors
DTR: Diphtheria toxin receptor
ECA: External carotid artery
PBS: Phosphate-buffered saline
ssGSEA: Single-sample gene set enrichment analysis
GSVA: Gene set variation analysis
ICC: Interclass correlation coefficient
BMT: Bone marrow transplantation
MACS: Magnetic activated cell separation
i.c.v.: Intracerebroventricular injection
ANOVA: One-way analysis of variance
DCs: Dendritic cells
cDCs: Conventional dendritic cells
pDCs: Plasmacytoid dendritic cells
CNS: Central nervous system
EAE: Experimental autoimmune encephalomyelitis
NSAIDs: Nonsteroidal anti-inflammatory drugs
