# Supplementary figures and images for: Ly6C-high monocytes alleviate brain injury in experimental subarachnoid hemorrhage in mice
Source: J Neuroinflammation. 2023 Nov 17;20:270. doi: 10.1186/s12974-023-02939-y (PMC10657171; doi:10.1186/s12974-023-02939-y)

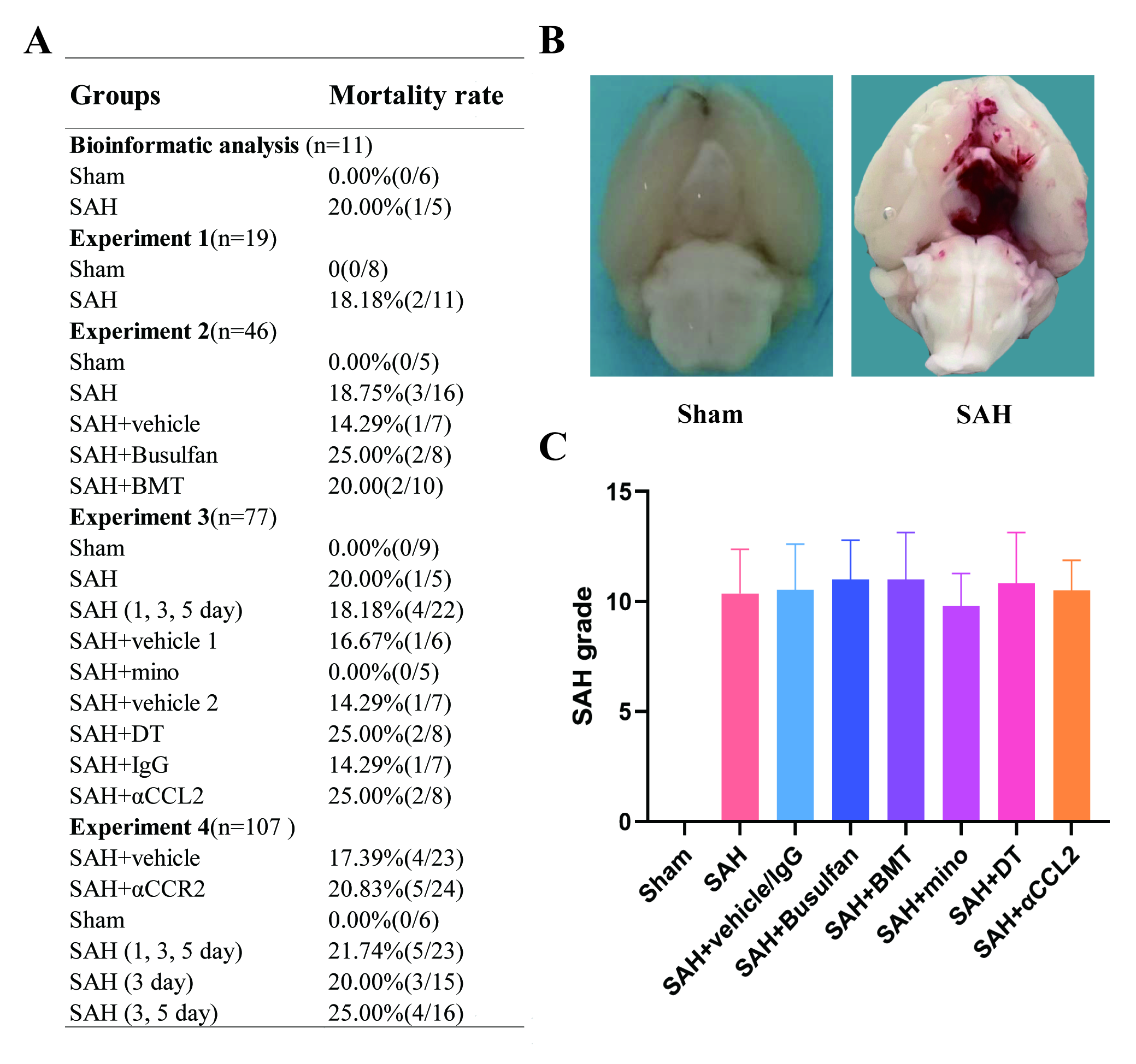

Supplement: Supplementary file 1 — Additional file 1: (A) Study groups and number of mice in each group. (B) Representative images of brains harvested from the sham and SAH mice. (C) SAH grade of each group in the study. [file 12974_2023_2939_MOESM1_ESM.tif]

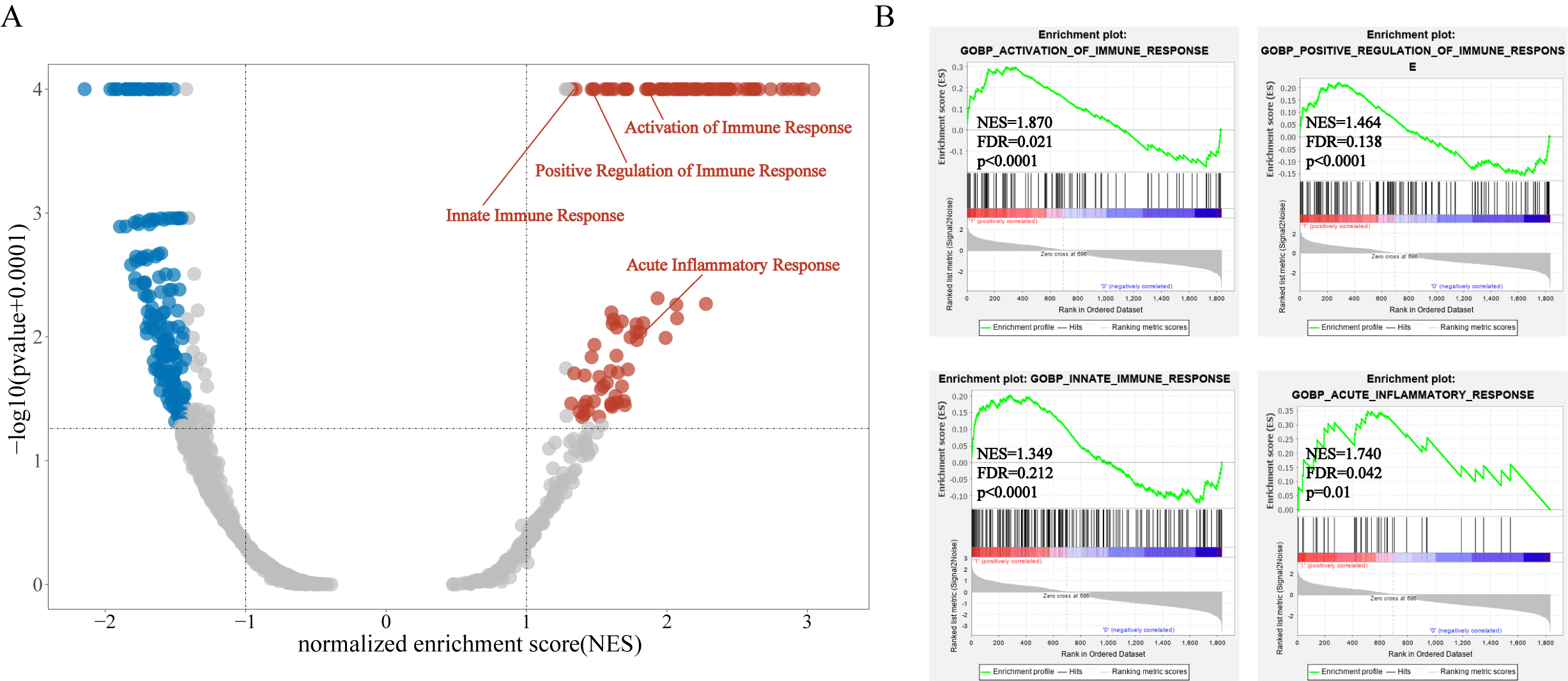

Supplement: Supplementary file 3 — Additional file 3: (A) Volcano plots showing upregulated and downregulated pathways in PXD030593. The upregulated biological processes were defined as those with NES > 1, p value < 0.05, and FDR < 0.25, whereas the downregulated biological processes were defined as those with NES < -1, p value < 0.05, and FDR < 0.25. (B) Plots showing the enriched pathways including activation of the immune response, positive regulation of the immune response, innate immune response, and acute inflammatory response in PXD030593. [file 12974_2023_2939_MOESM3_ESM.tif]

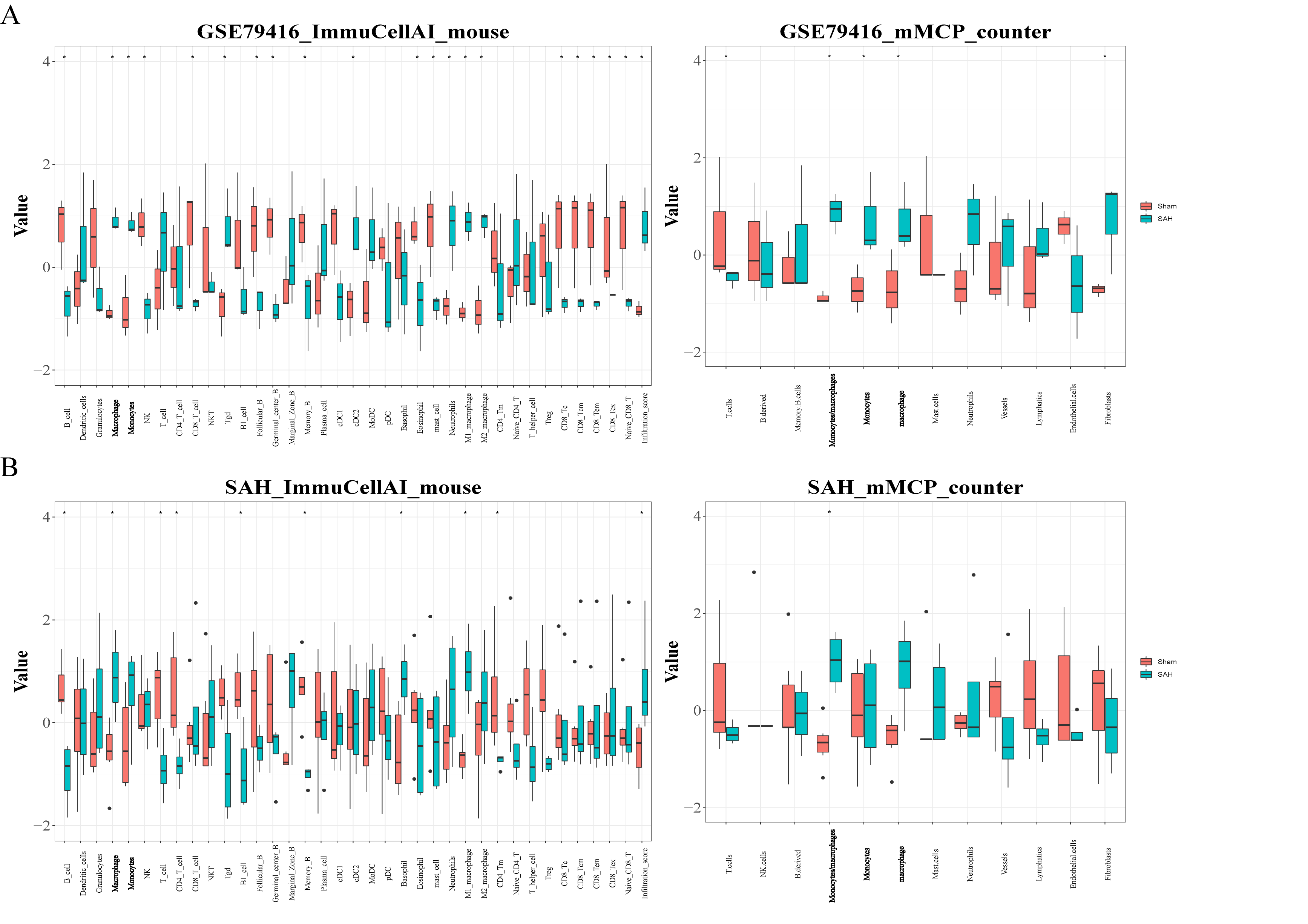

Supplement: Supplementary file 4 — Additional file 4: Bar plots showing the constitution of immunocytes in GSE79416 (A) and our data (B) calculated using ImmuCellAI_mouse and mMCP_counter. [file 12974_2023_2939_MOESM4_ESM.tif]

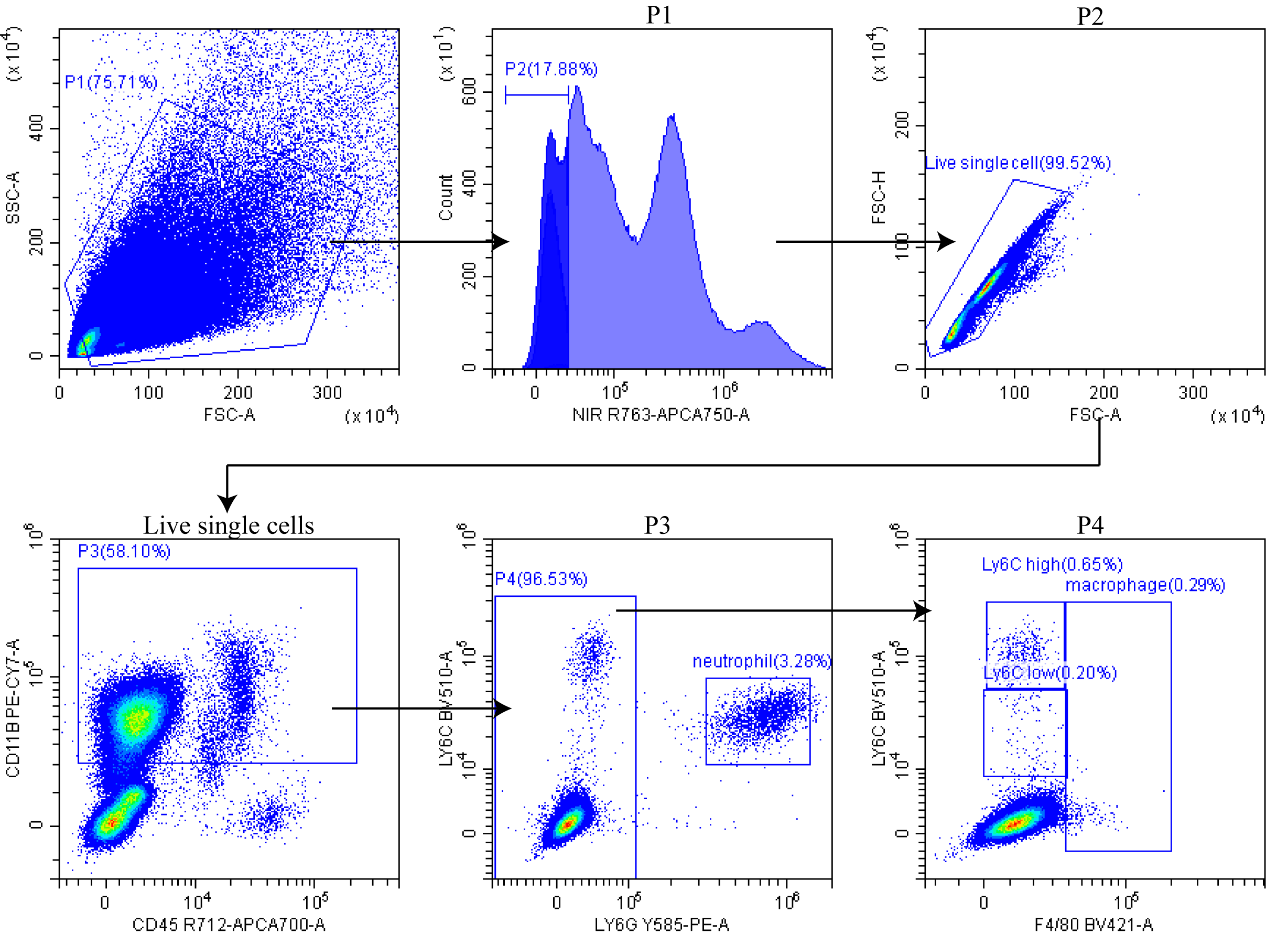

Supplement: Supplementary file 5 — Additional file 5: Gating strategy for Ly6C-high monocytes (CD45highCD11b+Ly6G−Ly6ChighF4/80−), Ly6C-low monocytes (CD45highCD11b+Ly6G−Ly6ClowF4/80−), and macrophages (CD45highCD11b+Ly6G−F4/80+). [file 12974_2023_2939_MOESM5_ESM.tif]

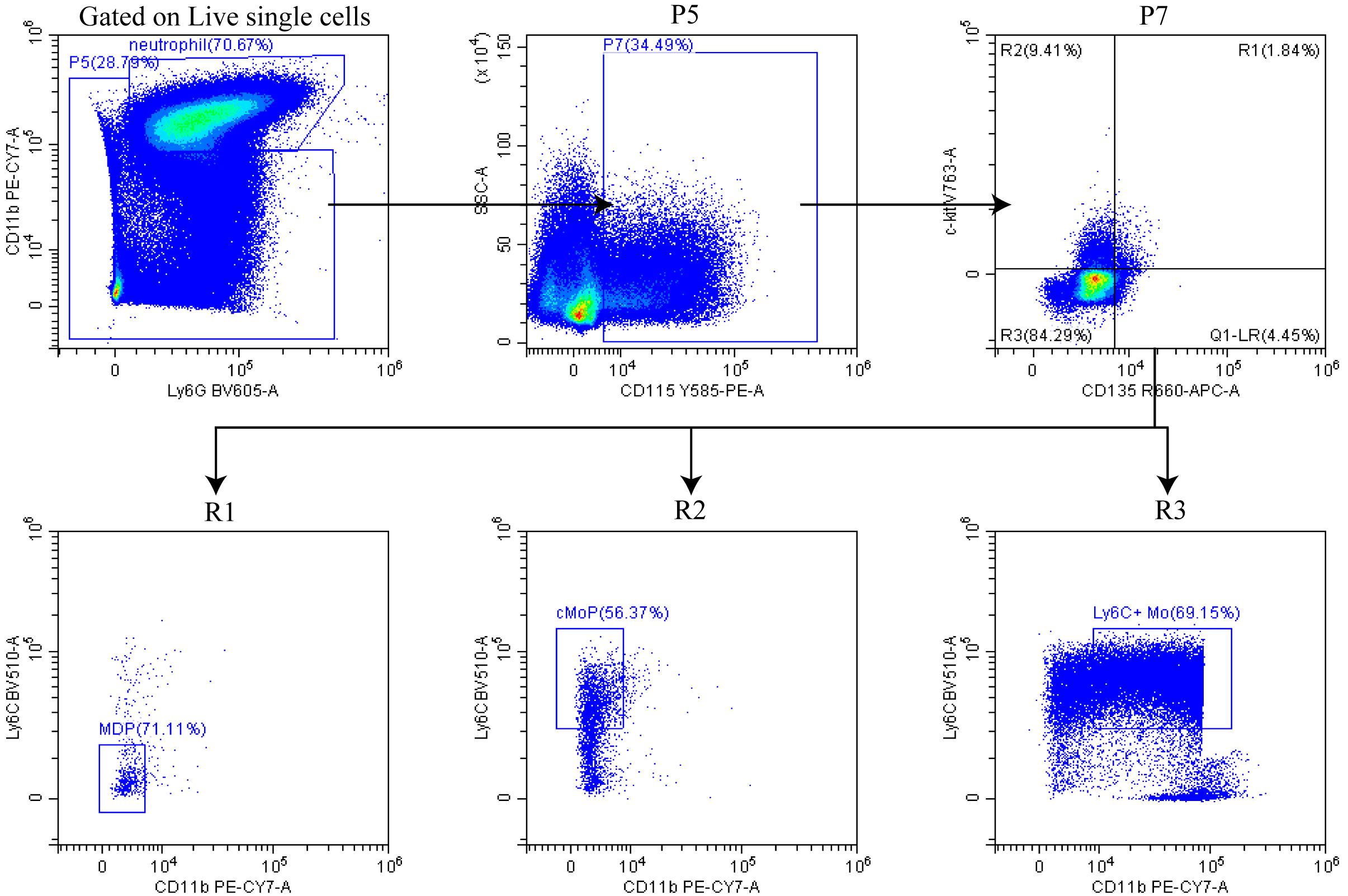

Supplement: Supplementary file 6 — Additional file 6: Gating strategy for MDPs (R1, CD115+CD135+c-kit+Ly6C−CD11b−), cMoPs (R2, CD115+CD135−c-kit+Ly6C+CD11b−), and Ly6C-high monocytes (R3, CD115+CD135−c-kit−Ly6C+CD11b+) in the bone marrow. [file 12974_2023_2939_MOESM6_ESM.tif]

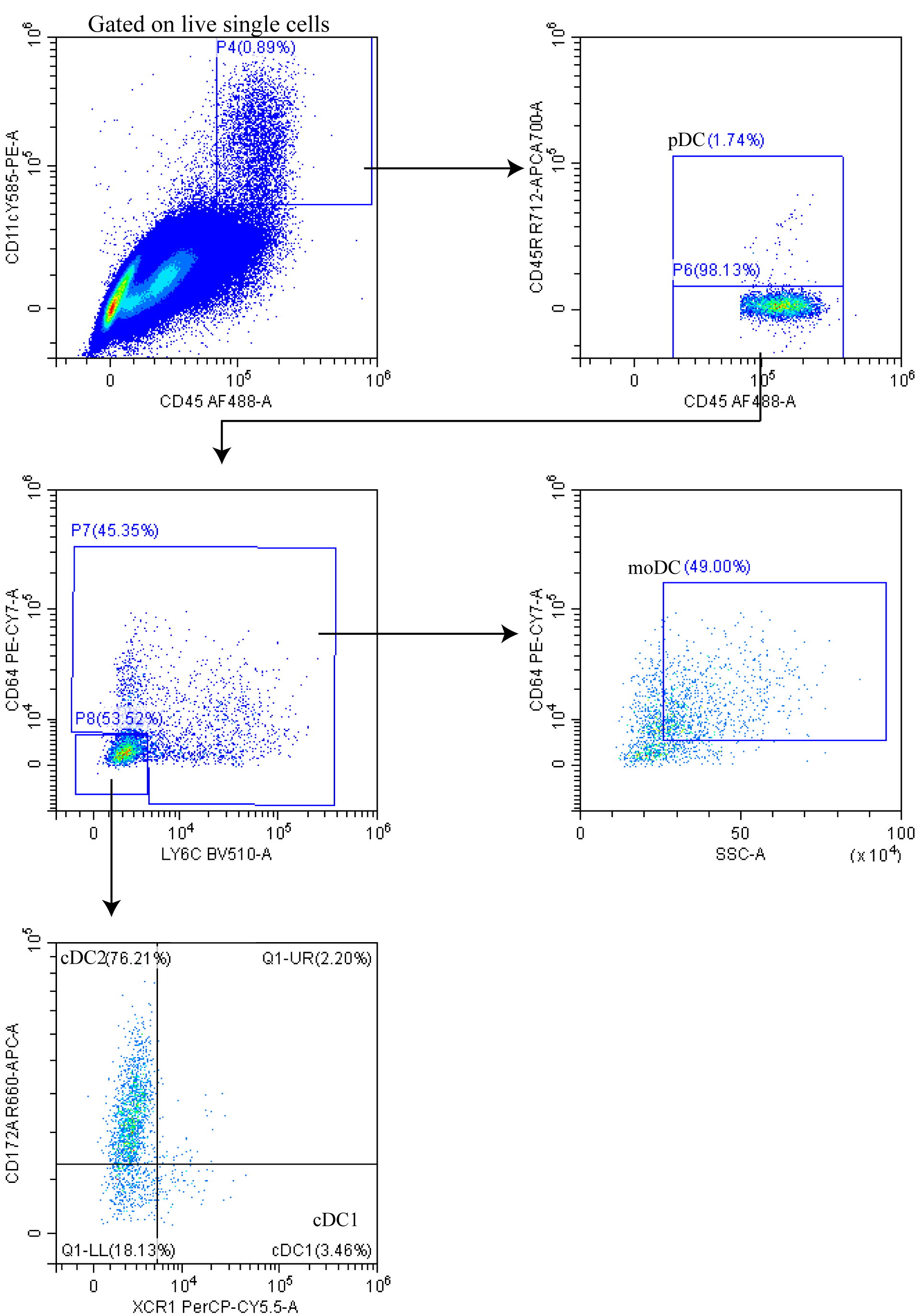

Supplement: Supplementary file 7 — Additional file 7: Gating strategy for pDCs (CD45highCD11c+CD45R+), cDC1 (CD45highCD11c+CD45R−Ly6C−CD64−XCR1+CD172A−), cDC2 (CD45highCD11c+CD45R−Ly6C−CD64−XCR1−CD172A+), and moDCs (CD45highCD11c+CD45R−CD64+). [file 12974_2023_2939_MOESM7_ESM.tif]
